# First Camera Trap Detection of a Gray Wolf Movement Into Nevada

**DOI:** 10.1002/ece3.71422

**Published:** 2025-05-08

**Authors:** Sean M. Sultaire, Robert A. Montgomery, Patrick J. Jackson, Joshua J. Millspaugh

**Affiliations:** ^1^ Wildlife Biology Program W.A. Franke College of Forestry and Conservation, University of Montana Missoula Montana USA; ^2^ Institute of Science and Environment University of Cumbria Ambleside UK; ^3^ Nevada Department of Wildlife Reno Nevada USA; ^4^ Department of Environmental Biology State University of New York, College of Environmental Science and Forestry Syracuse New York USA

**Keywords:** camera trap, *Canis lupus*, dispersal, gray wolf, Great Basin, large carnivore expansion, Nevada, wildlife monitoring

## Abstract

Following gray wolf ( *Canis lupus* ) reintroduction to Yellowstone National Park and central Idaho, USA, in the mid‐1990s, the species range has expanded into western Montana, eastern Oregon, and eastern Washington. By 2011, wolves reached northern California and formed multiple packs within a decade of their arrival in the state. Gray wolf observations have been sporadic, however, in the comparatively open and nonforested ecosystems such as the nearby northern Great Basin. During a broad‐scale, camera‐trapping study, we detected a gray wolf on an unbaited camera trap in northwest Nevada. This observation represents the 2nd confirmed sighting of a gray wolf in the state of Nevada since the 1920s and the first documented camera trap detection for the species in the state. We discuss this observation in the context of historical gray wolf presence in Nevada and the potential for the species to establish in the northern Great Basin.

## Graphical abstract

This photo of a gray wolf, captured in northern Nevada during a camera trapping survey, represents the second recorded sighting of the species in the state since extirpation. Wolf recolonization in western North America has generally followed forest cover and it remains undetermined whether the species can establish in steppe dominated regions like northern Nevada.

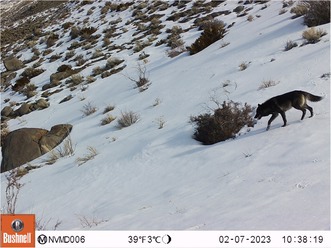

## 1 Introduction

The abundance and distribution of most species of large carnivores are declining worldwide in response to human persecution, overhunting of prey species, and habitat loss and degradation from the rapidly expanding human footprint (Ripple et al. 2014). Given their position towards the top of trophic systems, the loss of large carnivores can trigger cascading effects that have implications for ecosystem processes and the structure and composition of mammalian, avian, and plant communities (Estes et al. 2011). Accordingly, restoring large carnivores across their historical ranges, although challenging and controversial, has become a primary goal among wildlife managers and ecologists.

After gray wolf ( *Canis lupus* ) extirpation from the western U.S. in the 1920s, the species was reintroduced to Yellowstone National Park and central Idaho in the late 20th century (Fritts et al. 1997). Thereafter, gray wolf populations expanded into forested areas of Montana, eastern Oregon, and eastern Washington (Oakleaf et al. 2006; Carroll et al. 2012). Eventually, wolves reached northern California in 2011 (Nickel and Walther 2019), where at least 6 packs were established as of early 2025 and several dispersing individuals were detected capable of establishing in new areas (California Department of Fish and Wildlife 2025). Despite gray wolves occurring in a diversity of habitats globally and the contiguous U.S. historically, wolf pack recolonization in the western U.S. has largely been constrained by the availability of forest cover (Oakleaf et al. 2006; Mech 2017). For example, gray wolves have yet to establish in more arid ecosystems such as the northern Great Basin.

**FIGURE 1:**
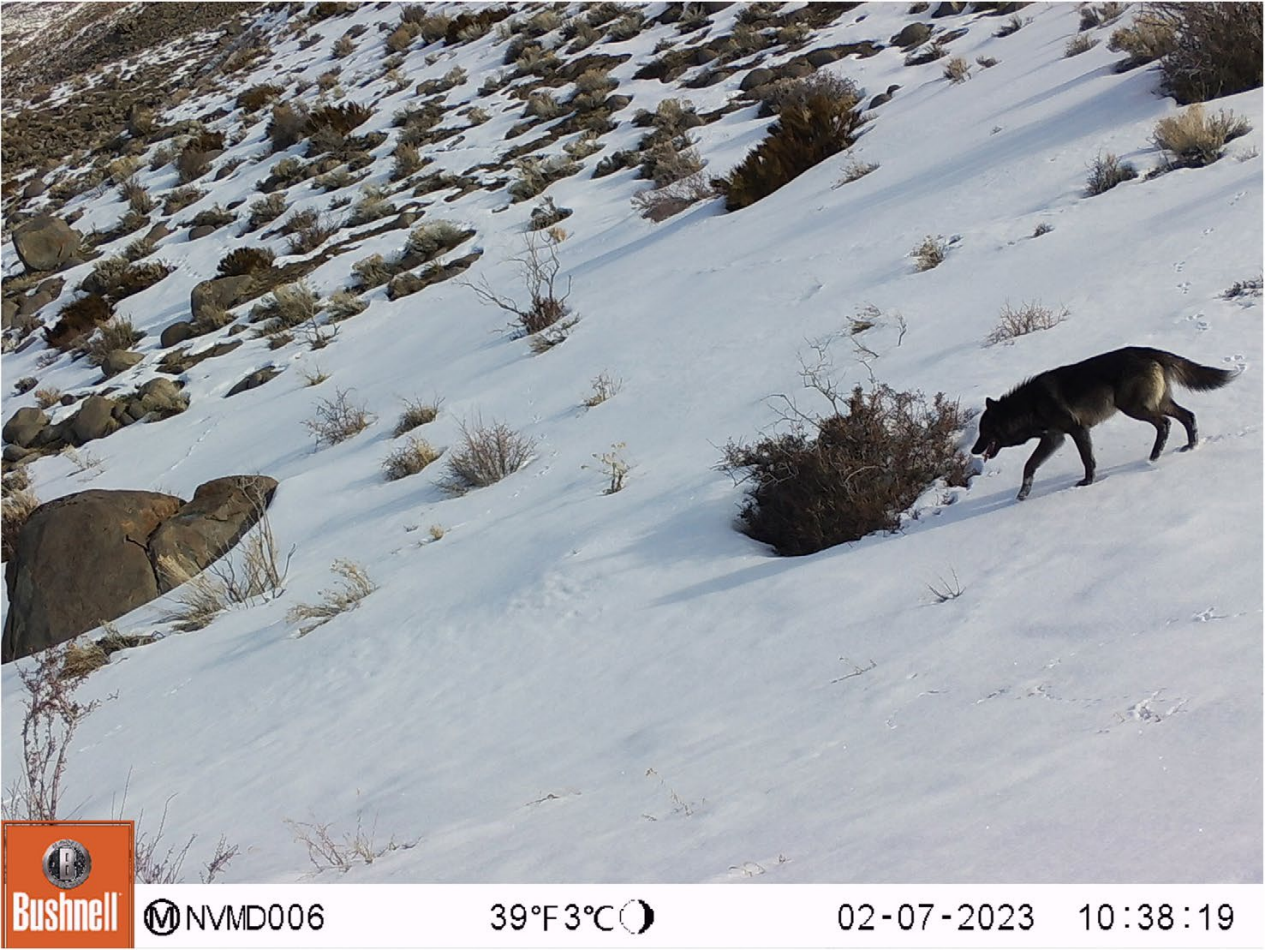
The first of three camera trap photographs in a sequence that captured a gray wolf ( *Canis lupus* ) in northwest Nevada in February 2023. This photo represents the 2nd verified gray wolf sighting in Nevada since the 1920s.

**FIGURE 2:**
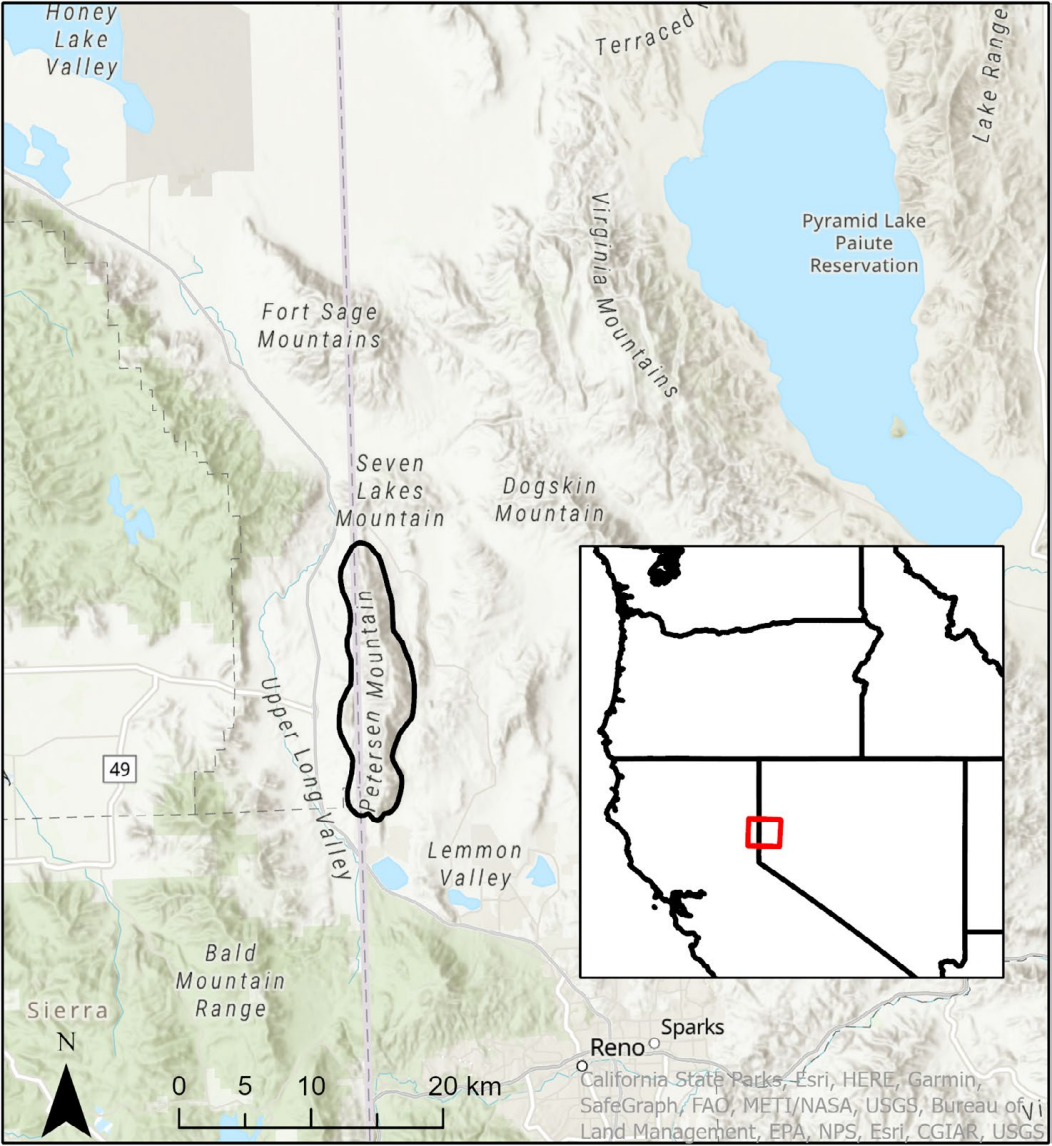
Map showing the location of the gray wolf ( *Canis lupus* ) we detected with a camera trap in February 2023 on Petersen Mountain (back oval), in northwest Nevada near the border with California. This region coincides with the ecotone between the Sierra Nevada and Great Basin Desert, with the green area coarsely representing the distribution of forest cover and white indicating open habitats, primarily sagebrush steppe.

On February 7th, 2023, we captured a photo of a gray wolf with black pelage in northwestern Nevada (Figure 1) on a camera trap located on Petersen Mountain, at an elevation of 1790 m (Figure 2). The location was approximately 30 km northwest of Reno, Nevada, and 1 km east of the border with Lassen County, California. The vegetation there is characteristic of Petersen Mountain, consisting of montane shrub steppe, with bitterbrush ( *Purshia tridentata* ), desert peach ( *Prunus andersonii* ) and nonnative crested wheatgrass ( *Agropyron cristatum* ). Small areas of juniper woodland (*Juniperus* sp.), with isolated Jeffrey ( *Pinus jeffreyi* ) and Ponderosa pines ( *Pinus ponderosa* ) were present nearby, but overall, forest and woodland only comprised 5% of the land cover on Petersen Mountain (LANDFIRE: LANDFIRE Existing Vegetation Type Layer 2019). Other mammals detected on this camera since December 2020 included mule deer ( *Odocoileus hemionus* ), coyote ( *Canis latrans* ), bobcat ( *Lynx rufus* ), and feral horse ( *Equus ferus* ). The closest wolf pack in California at the time of the detection and where this individual might have originated was the Beckwourth Pack in Plumas County, approximately 25 km to the northwest (territory edge). The camera was facing north, and the wolf was moving northwest towards the California border when detected. This camera was deployed as part of a regional‐scale study that included more than 200 cameras in western Nevada, from Reno north to the Oregon border, but no bait or lure was applied to attract wildlife to the camera.

This detection represents just the 2nd confirmed sighting of a gray wolf in Nevada since the 1920s, the third recent record of the species in the state (one GPS location, see below), and the first recorded camera trap detection for the species in the state. The previous contemporary gray wolf sighting in Nevada consisted of a video observation from November 2016, later confirmed through fecal DNA by the Nevada Department of Wildlife (Spillman 2017). This observation also occurred in the northwest part of the state near Fox Mountain, at approximately 284,904 E, 4, 544,725 N, which is 145 km northeast of our camera‐trap detection. Furthermore, in January 2021, a wolf that was GPS‐collared in Oregon briefly entered northwest Nevada from California near Verdi (~30 km south of our observation) and later Topaz Lake, prior to moving back into California (Patrick Jackson, personal obs.). This individual, however, was not directly observed or photographed.

Despite these recent detections, considerable ambiguity exists with regard to the historical extent of gray wolf range in Nevada and California (Shelton and Weckerly 2007). The only verified historical record for the species in Nevada was located in the northeastern corner of the state in 1922, with an additional unverified record (no specimen) from northwest Nevada in the 1910s (Hall 1946). Although a lack of historical reports in the Great Basin may be attributed to other factors, such as misidentification as coyotes, wolves appear to have been uncommon in the region preceding their extirpation in the early 20th century. Low densities of suitable prey may explain wolf scarcity historically; for example, elk ( *Cervus canadensis* ) were not present in the western Great Basin, and mule deer may have been uncommon (Berger and Wehausen 1991). However, ungulate species composition has shifted substantially in the Great Basin since European settlement, with mule deer likely more abundant (Berger and Wehausen 1991) and feral horses considered overabundant (Beever 2003; Coates et al. 2021; Stoner et al. 2021). Based solely on deer, estimates of prey biomass for the area (0.19 deer/km^2^; Sultaire, S.M. unpublished data) are below those reported elsewhere (0.73–12 deer/km^2^; Kuzyk and Hatter 2014), but feral horses are preyed upon by wolves elsewhere (Parr and McCrory 2022) and could serve as alternative prey. Increases in the biomass of these prey species could favor wolf establishment in the northern Great Basin, especially given evidence that cougars ( *Puma concolor* ), a competing large carnivore, have increased in the Great Basin since European settlement (Berger and Wehausen 1991; Sweitzer et al. 1997).

Although northern Nevada has atypical habitat compared to areas wolves have recently recolonized in the western U.S. (e.g., lower forest cover; Oakleaf et al. 2006), proximity to established packs in California suggests that wolves are likely to disperse regularly into the northwest Great Basin. Even if Nevada proves to be low‐quality habitat, wolf movement in the region could facilitate connectivity between wolf populations in California and those in the Rocky Mountains and southwest U.S. Our observation and previous detections of the species in northwest Nevada indicate that wildlife managers should closely monitor the area for signs of wolf pack establishment. Since this detection, two more wolf packs have established in this area in nearby California (California Department of Fish and Wildlife 2025), providing more potential source packs from which a Nevada colonization could originate. This gray wolf detection further emphasizes the value of broad‐scale camera trap networks (~17,000 km^2^) for passively detecting long‐distance movements and potential range expansion of large carnivores.

## Author Contributions

**Sean M. Sultaire:** conceptualization (equal), visualization (lead), writing – original draft (lead). **Robert A. Montgomery:** conceptualization (equal), writing – original draft (equal), writing – review and editing (equal). **Patrick J. Jackson:** conceptualization (equal), writing – original draft (supporting), writing – review and editing (supporting). **Joshua J. Millspaugh:** conceptualization (equal), writing – original draft (equal), writing – review and editing (equal).

## Conflicts of Interest

The authors declare no conflicts of interest.

## Data Availability

The authors have nothing to report.
